# Atomic force nanorheology for *in situ* exploration of nanoscale SEI layers in real-life rechargeable-battery electrodes

**DOI:** 10.1039/d6fd00053c

**Published:** 2026-04-23

**Authors:** Mangayarkarasi Nagarathinam, Yue Chen, Oleg Kolosov

**Affiliations:** a Physics Department, Lancaster University Lancaster UK o.kolosov@lancaster.ac.uk; b College of Physics and Energy, Fujian Normal University Fuzhou 350117 China

## Abstract

The solid electrolyte interphase (SEI) is a nanoscale thin layer with complex nanomechanical properties that separates a battery’s negative electrode and the electrolyte to allow free flow of active ions (Li or Na), while precluding electron flow. A robust and high-performance SEI is critical for battery performance, cyclability and safety, and the ability to study SEI properties in real-space and real-time (*operando*) holds the key for development of efficient and safe batteries. Here we report an efficient approach allowing probing of the nanoscale mechanical homogeneity of SEI layers with nanoscale depth resolution *via* scanning nanorheology microscopy (NRM). In NRM, the shear forces acting on the atomic force microscopy (AFM) nanoscale tip, measured as the tip penetrates the SEI layer from the electrolyte to the solid electrode surface, provide a 1D quantitative measure of local storage and loss elastic moduli of the SEI along the path of the tip. A key feature of the new 1D-NRM is the ability to probe nanoscale SEI dynamics *operando* in real-life battery electrodes that have micrometre-scale roughness, while providing nanometre-scale depth resolution. Significantly, we show that due to the nature of shear-force measurements, NRM effectively eliminates the dependence of the measurements on the diameter of the AFM tip, a parameter that is otherwise the hardest to quantify in AFM nanomechanical measurements. We successfully apply a new approach to quantify details of the SEI formation process on hard-carbon negative electrodes in Na-ion batteries.

## Abstract 2

The complex three dimensional nanoscale structure and nanomechanical properties of the SEI can be explored and quantified in real-life metal-ion battery electrodes *via* mapping shear forces acting on the AFM tip penetrating SEI layers.

## Graphical abstract

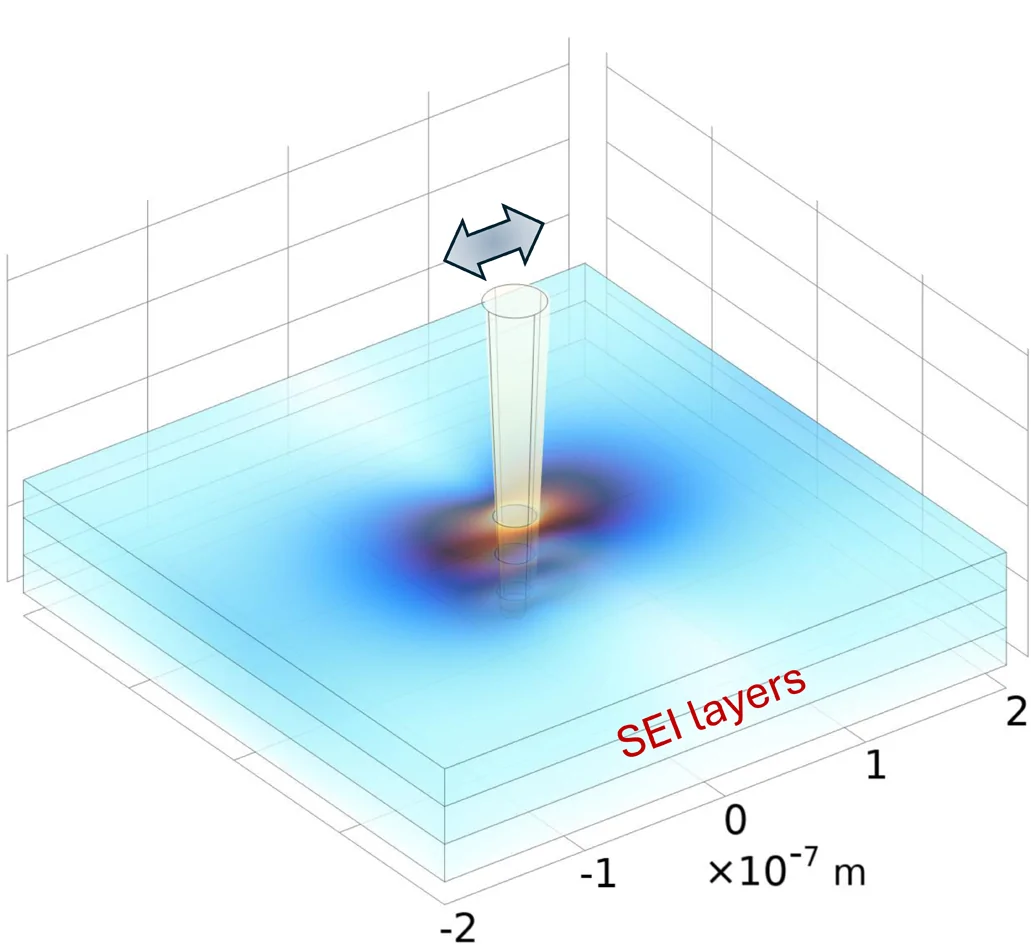

## 1 Introduction

If one were asked to single out the most critical component in non-aqueous rechargeable batteries, including lithium-ion, sodium-ion and next-generation emerging metal-based batteries, it is likely to be a solid electrolyte interphase, or SEI.^1^ In batteries, an SEI is a passivation layer a few tens of nanometres thick that forms at the negative electrode–liquid electrolyte interface during the initial chemical or electrochemical reduction of the electrolyte. At the initial cycling stage – often referred to as “formation” – SEI growth results in irreversible capacity loss and hence a drop in coulombic efficiency. During storage and cycling operation, the battery components (positive and negative electrodes, electrolytes) further decompose, and the initially formed SEI evolves, which eventually leads to capacity fade and power fade. As this dynamic character of the SEI results in the continuous consumption of electrolyte and active metal ions from the system, the SEI is one of the major contributors in both efficient battery operation over multiple cycles and battery degradation.^1^ In a nutshell, SEI formation at the electrode–electrolyte interface in rechargeable batteries is unavoidable. To extend battery life and performance, it is vital to develop a stable and thin initial SEI, which can effectively provide transport of ions to the electrode while acting as an effective barrier for the electrons, thus preventing further electrolyte decomposition and interfacial restructuring.

The SEI is composed of organic and inorganic components of crystalline, amorphous and polymorphic phases as well as trapped gases, electrolytes and salts.^2^ This mix results in complex nanomechanical properties of the SEI, ranging from a viscous solvent-like soft outer region to a mixed organic and inorganic transition layer with moderate stiffness and a stiff inorganic inner layer adjacent to the electrode. These properties evolve dynamically under real-time battery operating conditions and can be effectively described *via* the “viscoelastic”, or rheological, concept, where storage elastic moduli (such as Young's and shear moduli) are coupled with the loss moduli, the viscous response associated with the flow of the material, such as in liquids.^3–5^ The viscoelastic properties of the SEI play an important role in accommodating volume expansion in Li-ion batteries, suppressing lithium dendrite formation, with compromised mechanical stability of interphases leading to catastrophic failure in batteries.^6^ These factors call for novel real-space nanoscale resolution methods for measurement of an SEI’s physical proportions that match the nanoscale dimensions of the SEI and its heterogeneous components. Significantly, such SEI evaluation has to be performed *in situ* with the electrolyte in contact with the electrode and, more preferably, under real-time, or *operando*, conditions when the battery is being charged or discharged.^7–9^

Current techniques for mapping of the real-space and real-time properties of an SEI include optical microscopy, for which the lateral resolution is unavoidably limited by the sub-micrometre scale of visible wavelengths of light.^10,11^ Scanning electron and transmission electron microscopies do provide the required nanoscale spatial resolution; however, they require high vacuum and are therefore not compatible with the liquid electrolytes used in typical batteries. Recent advances in *operando* TEM/STEM, particularly using liquid-cell configurations, have enabled direct visualization of interfacial evolution under electrochemical conditions, reflecting structural, compositional and mass-thickness variations.^12^ However, such approaches remain limited in probing nanomechanical properties. In contrast, atomic force microscopy (AFM), which is able to measure local physicochemical properties, *e.g.* nanomechanical or electrical properties, with nanoscale resolution, provides an alternative approach that can overcome these limitations, enabling direct space-nanoscale studies of SEIs in realistic electrochemical environments.

AFM is a scanning probe microscopy technique that employs a nanoscale sharp tip to map material surfaces *via* tip–surface force interaction, and it can be operated in liquid and electrochemical environments. By providing topographic images of electrode surfaces, AFM has been shown to effectively visualize electrode degradation, metallic plating, and SEI growth.^13–16^ By employing nanomechanical AFM modes such as force modulation (FM), quantitative nanomechanical mapping (QNM), force–volume (FV)^2,17–19^ and ultrasonic force microscopy (UFM) modes,^20–22^ AFM can allow observation of the changes in the local mechanical moduli of relatively stiff regions, such as inorganic components of the SEI, directly on the electrode surface. However, these nanomechanical AFM techniques are inherently more sensitive to elastic or moderately stiff materials, while mapping and quantifying nanomechanical properties of highly viscous or liquid-like regions of the SEI remain challenging due to the dissipative and time-dependent response of such layers.

Recently, our group has demonstrated that it is possible to explore the whole thickness of a nanoscale SEI, from the outer viscous-like layer to inner stiff layers, using interactive measurement of the local shear moduli of the SEI on a model graphite surface as the nanoscale sharp AFM tip penetrates the SEI layer. This approach was called three-dimensional (3D) nano-rheology microscopy (3D-NRM).^23,24^ While 3D-NRM allows the construction of 3D maps of the SEI with a lateral resolution of ∼20 nm and vertical resolution below ∼5 nm, while simultaneously mapping local mechanical properties across the layered structure, these measurements were performed on an extremely simplified system of atomically flat graphite layers with roughness of sub-nm scale. At the same time, all realistic electrodes in batteries are not atomically flat materials and consist of grains that are sub-micrometre to a few micrometres in size with carbon black and polymeric binder, resulting in electrode roughness on the micrometre scale.

Here we present for the first time a modified NRM workflow that allows evaluation, with nanometre-scale depth resolution, the viscoelastic properties of real-life electrodes’ SEIs. The key component of this new approach is dynamic referencing of the solid–solid point of contact between the probing tip and the electrode surface with nanometre-scale precision at each measurement location. At each location, consisting of an approximately 20 × 20 nm^2^ area, multiple measurements in adjacent nanoscale-spaced areas provide a quantitative measure of the average values and statistical spread for the elastic and viscous components of the SEI as a function of SEI height above the electrode surface. We call this method 1D-NRM, and while it does not directly reconstruct the full 3D image of the SEI, it does evaluate the lateral and vertical nanoscale inhomogeneity and nanomechanical properties of the SEI *operando* for any real-life micrometre-scale rough electrodes on the ∼20–50 nm lateral length scale and <5 nm vertical scale. In this paper, we outline the complete workflow of the 1D-NRM measurements, encompassing measurement setup, instrumental requirements, and data analysis and means for the efficient presentation of the resulting nanomechanical characterization. These results are supported by the finite element analysis (FEA) of the AFM-tip–SEI-layer interaction, to quantitatively extract the nanomechanical rheological properties of the SEI layer. Finally, we demonstrate the application of 1D-NRM for *operando* measurements of the SEI on realistic rough hard-carbon (HC) electrodes in Na-ion batteries half-cell configurations.

## 2 Why nanorheology microscopy (NRM) for SEI characterisation?

**Fig. 1:**
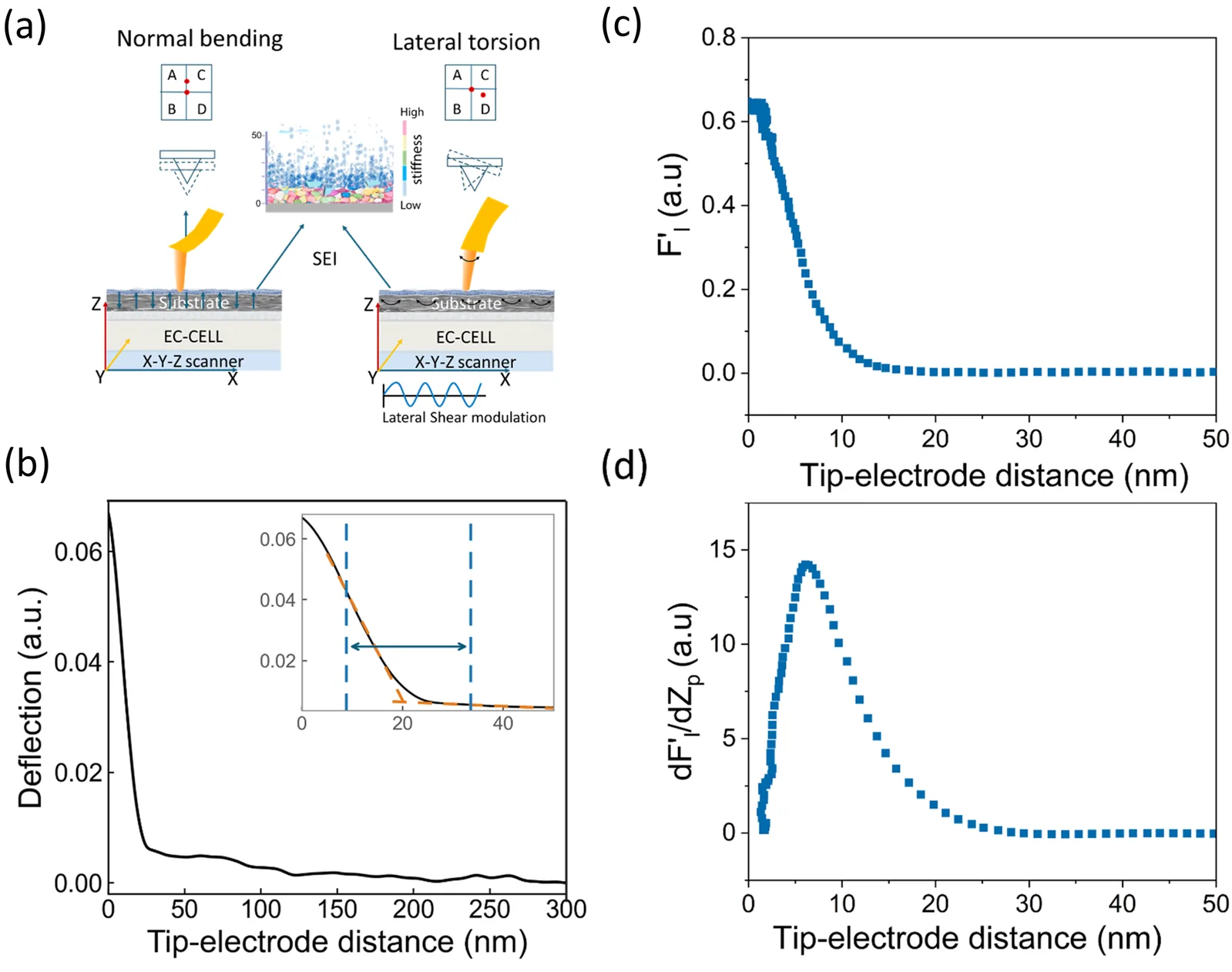
(a) Schematics of normal and lateral forces acting on the AFM tip and cantilever, (b) tip deflection *vs.* sample displacement (experimental example), inset – enlarged view of the transition region used to determine the onset of solid–solid tip–sample contact, (c) lateral force amplitude 
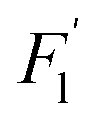
, (d) depth-resolved derivative 
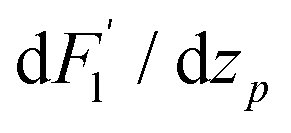
.

While multiple SPM techniques have been previously employed to probe a battery's interphase properties, they typically focused on the surface morphology of the SEI.^25^ At the same time, it is the nanomechanical properties that provide a more direct link to the local composition of the SEI. A typical SEI is mechanically heterogeneous and not only composed of stiffer crystalline inorganic components, but also of amorphous, soft and viscous organic and polymeric components that evolve continuously during cycling. Hence, there is a need for methodology that can capture these distinct nanomechanical regimes, from the viscous solvent-rich outer layer to the dense inorganic inner layer (Fig. 1). The existing nanomechanical AFM modes described above generally probe the force interaction between the apex of the AFM tip and the sample, making it difficult to probe the 3D structure of the SEI.^2,17–22^ At the same time, NRM that measures the lateral forces acting on the side of the AFM tip as it penetrates the internal structure is fully suitable for these studies.^24^ In NRM, the sample is oscillated laterally at a few hundred Hz to a few kHz frequencies at a very small (nm to sub-nm) amplitude that does not disrupt the SEI due to the vibration. It is important to distinguish that while the lateral oscillation amplitude is in the nm range, the penetration depth is controlled independently through the normal approach of the tip, enabling stepwise probing of the SEI with nanometre-scale resolution from the outer regions towards progressively stiffer domains. Also, rather than oscillating the tip and observing the damping of oscillation due to the tip–SEI interaction, NRM oscillates the sample, leading to practically zero response outside of contact with the SEI, providing a response that is directly proportional to the viscoelastic properties of the SEI. As the lateral response is not affected by the normal forces and is sufficiently large even for the relatively weak SEI gel components, NRM becomes directly suitable for studying the complex, relatively soft viscoelastic structure of the SEI layer, including both soft, viscous and progressively stiffer inorganic components. Importantly, by tracking the evolution of the lateral force response as a function of penetration depth, NRM enables continuous mapping of the nanomechanical properties across the SEI, from the viscous solvent rich-outer layers to the denser mechanically stiff inorganic regions closer to the electrode surface. This capability is fundamentally distinct from electron microscopy techniques, where contrast arises from mass–thickness variation rather than direct mechanical responses. For the purpose of studying the much stiffer inorganic component of the SEI on the near surface of the electrodes or SEI–electrode interface, and the electrode itself, UFM^22,26^ would be a better choice; however, this is outside the scope of the present work.

## 3 Normal and shear force measurements in lateral modulation AFM

AFM measures the forces acting between the apex of the nanoscale sharp tip positioned at the end of the flexible micromachined cantilever *via* its deflection. This deflection in modern AFMs is measured *via* either angular deflection of the laser beam reflected from the end of the cantilever or *via* optical interferometry.^27^ If only normal forces *F*_n_ acting along the tip axis are present, the deflected laser beam lies in the same plane as the incident laser beam and the cantilever long axis. By calibrating the flexural spring constant of the cantilever, the tip–surface normal force is then directly found *via* cantilever deflection. With the typical cantilever spring constants, *k*_c_, being in the range between 0.1 and 40 N m^−1^, and the deflection sensitivity in the range of a few pm, forces from a few pN to µN can be measured. The laser beam deflection angle, *Φ*, is proportional to the tip normal displacement and typically detected as the voltage, *V*_n_, so in order to link this to the tip displacement, the sample is brought in solid–solid contact with the tip and while the AFM sample is moved by the prescribed distance *d*_s_ with the piezo scanner, the ratio of this distance and the measured voltage *V*_n_ is recorded, providing absolute measurements of the tip deflection (Fig. 1b). By using a value of the spring constant of the cantilever, *k*_c_, provided by the manufacturer or calibrated *via* the thermal tune or Sader method,^28^ the absolute value of the tip-surface normal force, *F*_n_, can be measured.

During AFM operation, the tip is scanned in a raster manner across the surface using a three-axis piezo scanner to obtain the area image in the *x*–*y* plane, while the normal force is kept constant using a feedback loop by changing/adjusting the vertical position of the sample, *z*_s_, hence producing a topography image *z*_s_(*x*,*y*) of the sample with nanoscale lateral and vertical resolution. In addition to this normal interaction, the movement of the AFM tip across the sample surface also generates shear, also called lateral, forces. In a simple case, these forces are coming from solid–solid friction between the tip and the sample, which can also be modified by the omnipresent surface liquid layers under ambient conditions. If the scanning direction is perpendicular to the long cantilever axis, the lateral force *F*_l_ creates a torque *τ* = *l*_t_*F*_l_, where *l*_t_ is the length of the cantilever tip, resulting in torsion of the cantilever by the angle *θ* = *τ*/*k*_t_, where *k*_t_ is the torsional stiffness of the cantilever. The magnitude of this torsion hence depends on the lateral force, tip length, and torsional stiffness of the cantilever. The resulting torsion angle *θ* is detected from the out-of-plane (the plane defined by the incident laser beam and the cantilever long axis) deflection of the reflected laser beam using a quadrant light photodiode that registers voltage *V*_l_. The lateral torsion angle of the cantilever, *θ*, can be calibrated in a similar way as the normal response, by locking the tip in solid–solid contact with the sample and moving it laterally with the AFM piezo scanner by a prescribed value. The values of *l*_t_ and *k*_t_ can be directly derived from the geometrical parameters of the AFM cantilever-tip provided by the manufacturer, allowing direct evaluation of the lateral force as1
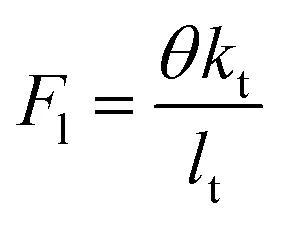


If the sample cannot be presented as a solid surface, such as in the case of a viscoelastic SEI layer with a thickness of several tens of nanometres, the situation changes dramatically. In this case, as the AFM tip penetrates into such a layer, additional forces emerge that act not only on the surface but also act along the side surface of the nanoscale tip, both along the parallel and perpendicular to the tip axis. As a result, the total lateral force, *F*_l_, acting on the AFM cantilever no longer solely represents only the surface interaction, but corresponds to the integral contribution of the forces encountered by the individual layers of the SEI as the depth of penetration, *z*_p_, of the tip increases (Fig. 1c). Here we assume that the penetration depth of the tip into the SEI (typically <100 nm) is at least two orders of magnitude smaller than the length of the tip (10–20 µm), allowing the consideration that all lateral force is effectively applied to the apex region of the tip. However, despite this geometrical confinement, the measured lateral force *F*_l_(*z*_p_) does not purely represent a local interaction. Instead, as the tip penetrates into the SEI, it remains in contact with all the overlying layers it penetrated through. Therefore, the lateral force corresponds to the cumulative contribution of the material from the SEI surface down to the penetration depth, *z*_p_ (Fig. 1c). As a result, the direct measurement of *F*_l_(*z*_p_) provides the integrated force values down to the specific depth within the SEI. Then, by differentiating the *F*_l_ with respect to *z*_p_ (d*F*_l_/d*z*_p_), a quantitative parameter proportional to the rheological parameters of the individual layer at the specific penetration depth *z*_p_ can be obtained (Fig. 1d). In NRM, the sample is oscillated in the lateral direction in the *x*–*y* plane at a frequency *f* with an amplitude *A*_l_ to generate time-dependent lateral displacement *d*_l_(*t*) = *A*_l_ cos(2π*ft*), inducing a resulting lateral force response.

For viscoelastic materials such as the SEI, the response is phase shifted relative to the applied displacement and therefore represented as a complex quantity 
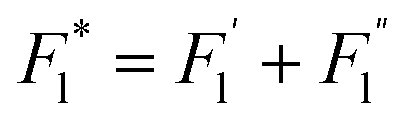
, where 
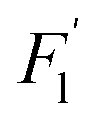
 and 
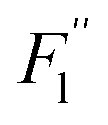
 represent the in-phase (elastic) and out-of-phase (viscous) components. As mentioned earlier, the depth-resolved derivative of this complex force, 
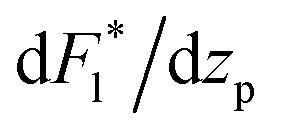
, provides the local force response associated with the material at depth *z*_p_ proportional to the amplitude of the applied oscillation. To calculate the material response that is independent of the excitation conditions, the depth-resolved derivative is then normalized by the oscillation amplitude *A*_l_, which can be represented as a complex value, the lateral sample compliance S*(*z*_p_) of the layer at the depth *z*_p_:2
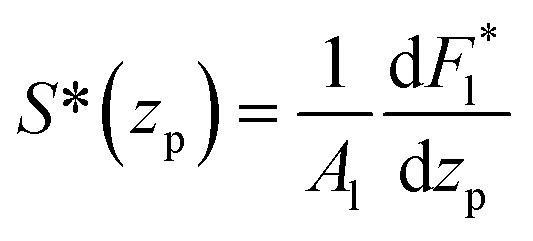


This parameter represents the local viscoelastic response of the SEI per unit lateral displacement, effectively characterizing the depth-resolved shear stiffness of the materials. It is to be noted here that the units of S*(*z*_p_) are N m^−2^, the same units as the Young's elastic modulus, *E*, of the material. Clearly the force response should be proportional to the complex shear modulus of the SEI at a particular depth, *Y*(*z*_p_).3
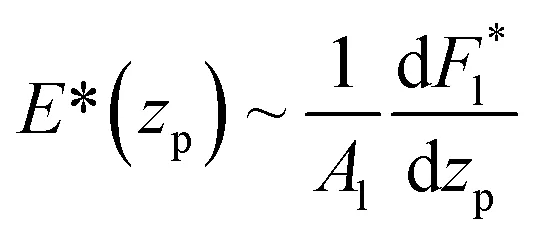


One can use reference materials with known elastic moduli or a finite element analysis (FEA) modelling to find the dimensionless coefficient of proportionality in eqn (3) and hence quantify the complex shear modulus of the SEI at a particular depth, *Y*(*z*_p_). The complex elastic modulus E*(*z*_p_), which corresponds to the viscoelastic property of the material, can be decomposed into the real part Re[E*(*z*_p_)], corresponding to the elastic (storage) component of the elastic modulus, and the imaginary part Im[E*(*z*_p_)], reflecting the viscous (flow component) behaviour of the SEI. The pathway for the quantification of elastic moduli is described in the next section.

## 4 Quantification of the shear rheological properties of layers *via* NRM

Here the step-by-step measurement methodology described in Section 3 is employed to obtain the calibrated values of in-phase and out-of-phase lateral forces acting on the tip, 
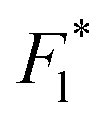
, as a function of the tip penetration depth *z*_p_. During these measurements, it is assumed that the sample is laterally dithered with the amplitude *A*_l_ producing time-dependent lateral displacement of the sample of *d*_l_(*t*) = *A*_l_ cos(2π*ft*) perpendicular to the long axis of the cantilever. Since the viscoelastic response of the material is determined from the phase difference between the applied displacement and measured force, it is essential to ensure that this phase shift originates solely from the sample and is not influenced by instrumental artefacts. In reality, the AFM scanner and actuator can introduce additional phase shifts, particularly near their resonance frequencies. For practical implementation it is therefore important to avoid the instrumental phase difference arising from actuator resonance. For example, if a sample-scanning AFM (Multimode, Bruker) is used, these resonances would typically be in the range of a few Hz to a couple of kHz. Even if shear transducers (*e.g.*, from PI ceramics) are used, for the sample-scanning AFMs used in our setup, the scanner is still expected to produce unwanted resonances. At the same time, the tip-scanning AFMs (such as Bruker ICON or Park NX-10) would not have the same limitations and dithering frequencies up to multiple tens of kHz can be used provided the sample itself is of a small mass. We also found that an amplitude of 0.5 to 2 nm of lateral dithering is more than sufficient for the effective mapping of the nanorheology of materials and provides sufficient sensitivity while maintaining a stable phase response and operation within the linear regime. Finally, linear (“diving board”) cantilevers with a relatively small spring constant (from 0.1 to 3 N m^−1^) are the best for NRM measurements due to the suitable torsional spring constant.

Once reliable experimental conditions and quantified lateral forces are measured, it is necessary to relate the measured lateral force response, 
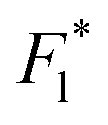
, to the intrinsic mechanical properties of the material. Since the NRM signal is based on the lateral force acting on the tip, a physical description of how this force depends on the material properties, tip geometry and penetration depth is required. Here we use FEA to estimate the additional geometrical coefficient at the local deformation level to account for the nonuniform strain distribution in the SEI near the apex area of the tip.

Firstly, let us estimate the forces acting on a tip of radius *r*_t_ with a length *z*_l_ embedded in the uniform viscoelastic layer. Here we will approximate the AFM tip as a cylinder with radius *r*_t_, which, given the typically small angle of the Si-based AFM tips we used, is a reasonable approximation that can be further rectified if higher accuracy is required. The area of the intersection of the tip and the layer perpendicular to the tip motion is then 2*r*_t_*z*_l_. Given that the amplitude of the normal displacement of the sample *A*_l_ (∼1 nm) is much smaller than the radius of the tip (∼10 nm), the resulting strain due to the lateral movement of the tip with respect to the sample can be estimated (subject to the dimensionless geometrical coefficient *r*_g_) as 
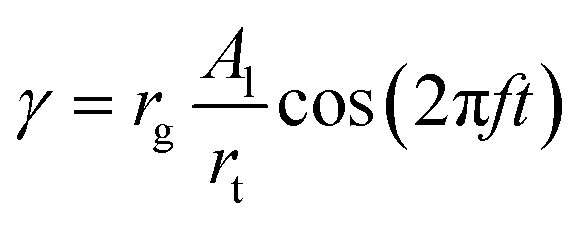
, leading to the average stress σ* = E*γ, where E* is the Young's modulus of the material. Then the average lateral force acting on the tip is the sum of the normal forces acting on the front and the back of the tip:4
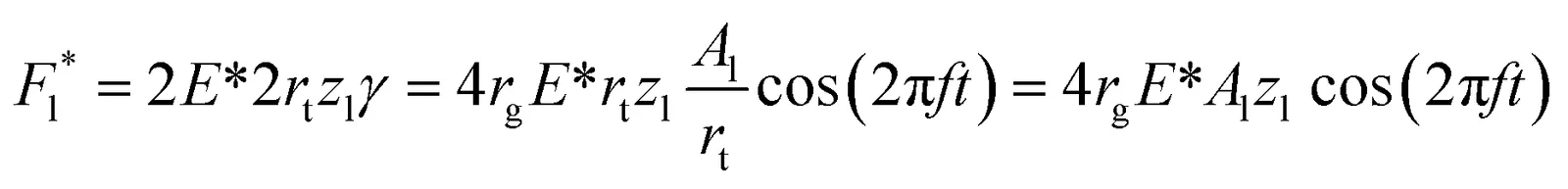


Significantly, eqn (4) shows that the dependence of the lateral force on the tip radius is fully eliminated, since the dependence of the strain is inversely proportional to the tip radius, while the area of contact experiencing the strain, and hence the force, is directly proportional to the tip radius. As the tip penetrates into the layer, the increment of the force (note that *z*_l_ = −*z*_p_), 
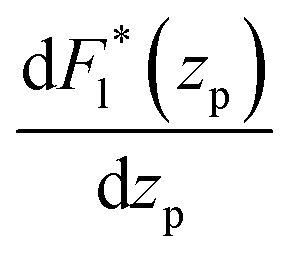
, is proportional to the Young's modulus at the depth *z*_p_, allowing the extraction of the effective Young's modulus of the layer as5
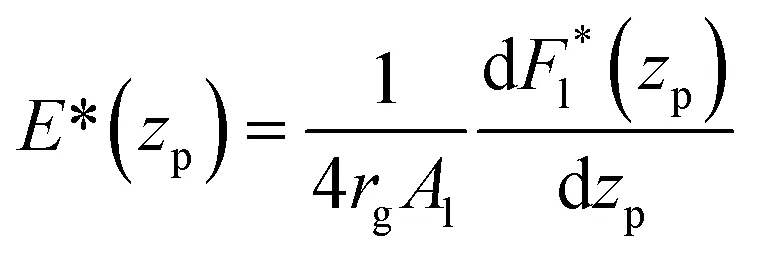


By recording the real (in-phase) and imaginary (out-of-phase) components of the force 
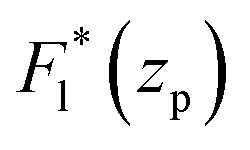
 as the AFM tip penetrates the SEI using a lock-in amplifier (SRS830, Stanford research instruments) and then differentiating with respect to *z*_p_, the storage *E*′ and loss *E*″ moduli (where E* = *E*′ + *iE*″) of the SEI material at depth *z*_p_ can be directly obtained.

Finally, for soft materials (gel-like, rubber, *etc.*) that can, with good approximation, be considered as non-compressible media, the Poisson's ratio, *ν*, becomes close to 0.5. In this case, the shear modulus becomes6
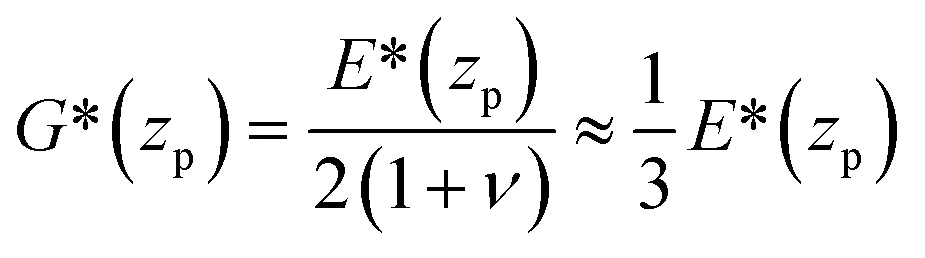


We then can link the real and imaginary part of the measured G* = *G*′ + *iG*″ with the position variable viscosity determined as7
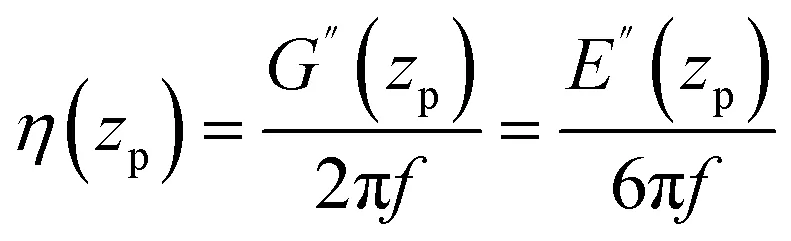


The dimensionless coefficient *r*_g_ is found as below using finite element analysis (FEA) simulation, by comparison of the simulated dependence 
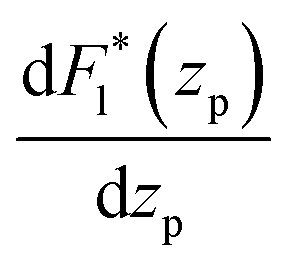
 for the homogeneous layer of the reference material with known Young's modulus *E*_ref_ under a known amplitude of the lateral sample *vs.* tip motion. In this case, the *r*_g_ is found as8
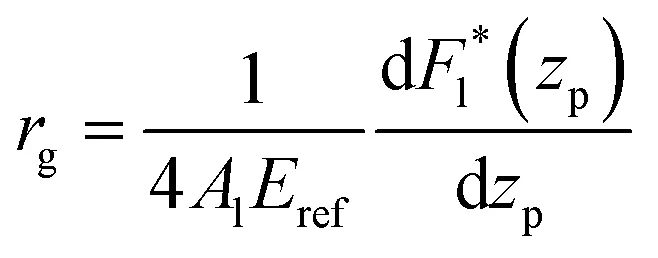


**Fig. 2:**
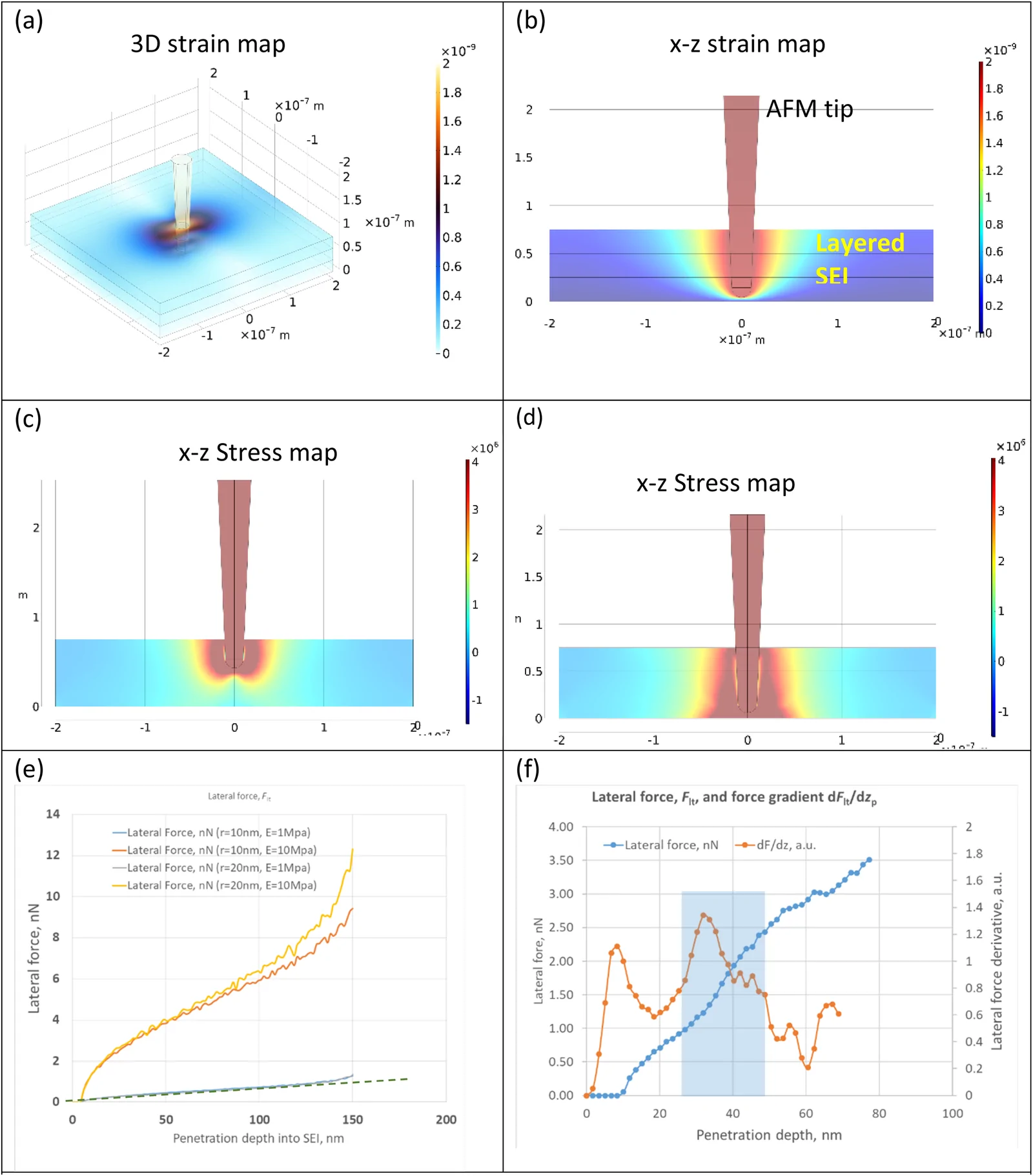
Finite element analysis (FEA) simulation of the lateral force in the NRM tip penetrating the SEI model layer. (a) 3D and (b) cross-plane view of the displacement amplitude in the SEI layer, (c) and (d) cross-sectional maps of the stress in the measured layer at different penetration depths (tip radius 10 nm). (e) Graphs of the lateral force for two layers with 1 MPa and 10 MPa Young's moduli and tip radii of 10 and 20 nm as a function of the penetration depth. Significantly, the response of NRM practically does not depend on the tip diameter, making the quantitative NRM approach possible without knowledge of the exact diameter of the AFM tip. The linear part of the penetration curve of the 1 MPa layer is used to find the dimensionless coefficient *r*_g_ = 1.25, which is necessary for the quantitative measurements of the Young's and hence shear moduli of the SEI as the function of the depth; deviation from linearity is expected as the tip–surface distance becomes comparable with the tip radius of 10 nm. (f) Simulation of the force and force derivative profiles for the SEI structure, with the internal stiff (“skin”) layer marked as a blue rectangle. The derivative of the force reliably picks up the inhomogeneity in the SEI internal nanostructure.

We compared the obtained values of E* and 
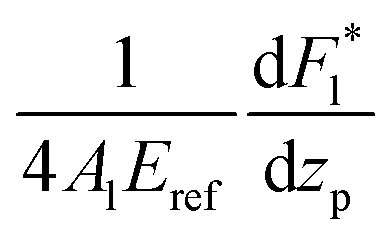
 to obtain *r*_g_ for the cylindrical tip. Fig. 2 shows the results of the simulation, showing the 3D and cross-section views of the displacement and stress and the calculated force *vs.* penetration (Fig. 2c), which allows calculation of the coefficient of *r*_g_ for a 2 nm lateral excitation amplitude *A*_l_, 1 kHz oscillation frequency, and a layer with 1 MPa and 3 MPa Young's moduli, as 
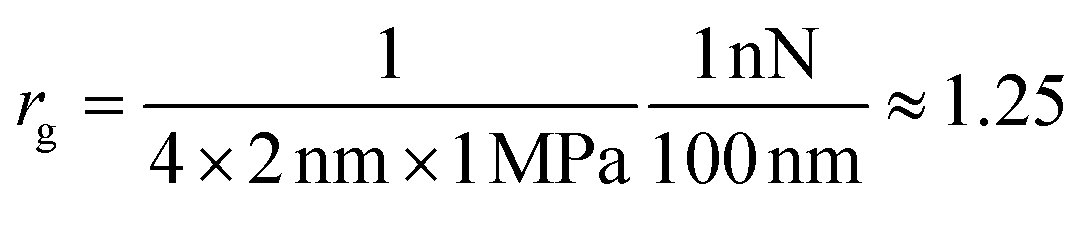
.

Notably, using exact simulations, we find that *r*_g_ is close to 1, validating our empirical estimates of the forces in the beginning of this section, and significantly, that the 1D-NRM does not require exact knowledge of the tip diameter, which in other nanomechanical modes is one of the most difficult experimental parameters to quantify, and which directly influences quantitative measurements in other, non-NRM nanomechanical modes.^2,17–22^

## 5 *Operando* mapping of SEI growth in the Na-ion system with real-life hard-carbon electrodes

**Fig. 3:**
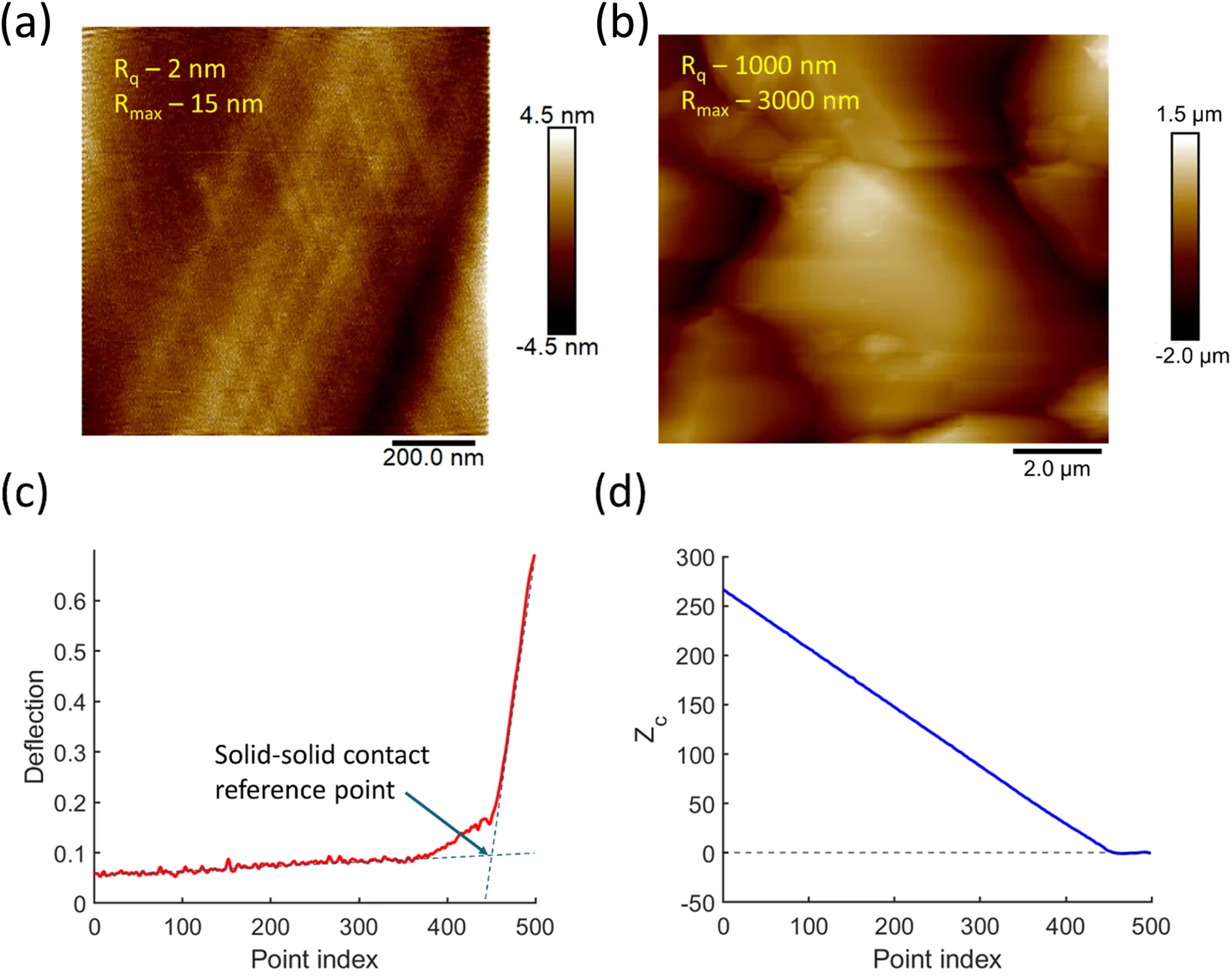
(a) HOPG and (b) HC negative electrode and their roughness values; (c) raw data of the deflection *vs.* sample displacement (measured in increment points) for the sample in (b) obtained in the measurement, in arbitrary units; the intersection of the base line and high-force solid–solid contact line defines the solid–solid contact point; (d) processed data with calibrated tip–electrode surface distance (calibrated vertical scale in nm), with solid–solid contact as the origin, as a function of the same increment steps.

The previous reports of the 3D-NRM approach for understanding nanomechanical properties used model electrodes, such as highly oriented pyrolytic graphite (HOPG) with atomic scale flatness and minimal surface roughness. Under these conditions, the electrode surface can be treated as a well-defined global reference, and this enables direct evaluation of the viscoelastic properties of the SEI at nanometre-scale spatial resolution. However, real battery electrodes, such as hard carbon, exhibit complex and heterogenous surface morphologies, with particle sizes ranging from hundreds of nanometres to micrometres, resulting in a surface roughness that is tens to hundreds of times larger than that of model electrodes (Fig. 3a and b). For example, the variation in morphology and surface roughness leads to differences in measurements, *i.e.*, at point A, the surface height is ∼0 nm and at point B, the surface height is ∼500 nm. Under such conditions, conventional global depth-referenced measurements become unreliable, as variations in topography obscure the true position of the electrode surface.

To address this limitation, here we focus on the ability to exactly pinpoint the position of the surface of the electrode and reference all nanorheological measurements to this point (Fig. 3c and d). Using this approach, multiple measurements were acquired over a large area (a few hundred nm to µm) with the lateral spacing on the order of nanometre-scale steps (∼50–100 nm) in a single statistical pool. Each measurement is locally referenced to the electrode surface by defining solid–solid contact as a zero tip–surface distance, even when the adjacent points can have micrometre-scale height differences. In order to do that, we considered that for the sample far from the tip, the normal force *F*_n_*vs.* sample position *d*_s_ ≫ 0 has a dependence *F*_n_(*d*_s_) = 0, and hence is flat, as there is no normal force acting on the tip. At the same time, once the tip touches the sample and therefore the sample pushes the cantilever up with *d*_s_ ≪ 0, in the limit of the high tip–surface force, the solid–solid contact is established and, in the tip, follows the sample surface, providing linear dependence of *F*_n_(*d*_s_). The intersection of these two lines – at *d*_s_ ≫ 0 and *d*_s_ ≪ 0 – is therefore used as a point of the solid–solid contact and is independent of the layers above the solid electrode surface. This procedure is automatically applied to each local measurement, allowing reliable determination of the ensemble of measurements referenced to the sample surface with nanometre-scale accuracy, even when the roughness of the sample is several orders of magnitude higher at the micrometre scale.

The core of this approach is shown in Fig. 3c and d, where the normal force displacement behaviour is used to determine the point of solid–solid contact for each individual measurement. When the tip is far from the electrode surface, *i.e.*, in the non-contact regime, the normal force remains negligible, whereas upon contact, the cantilever deflection increases linearly with sample displacement. The intersection of this regime defines the contact point and is assigned as the zero tip–surface distance (*z*_p_ = 0), as shown in Fig. 3c. This procedure is applied independently to each measurement location, which enables accurate reference of depth coordinates across regions with large topographical variation, as in Fig. 3b. By referencing all measurements to the locally determined surface position, this method enables the acquisition of statistically meaningful, depth-resolved nanorheological data across large areas of rough electrodes. The resulting aligned datasets form the basic data set for subsequent analysis including depth-dependent viscoelastic nanomechanical profiling and statistical evaluation of the SEI heterogeneity.

**Fig. 4:**
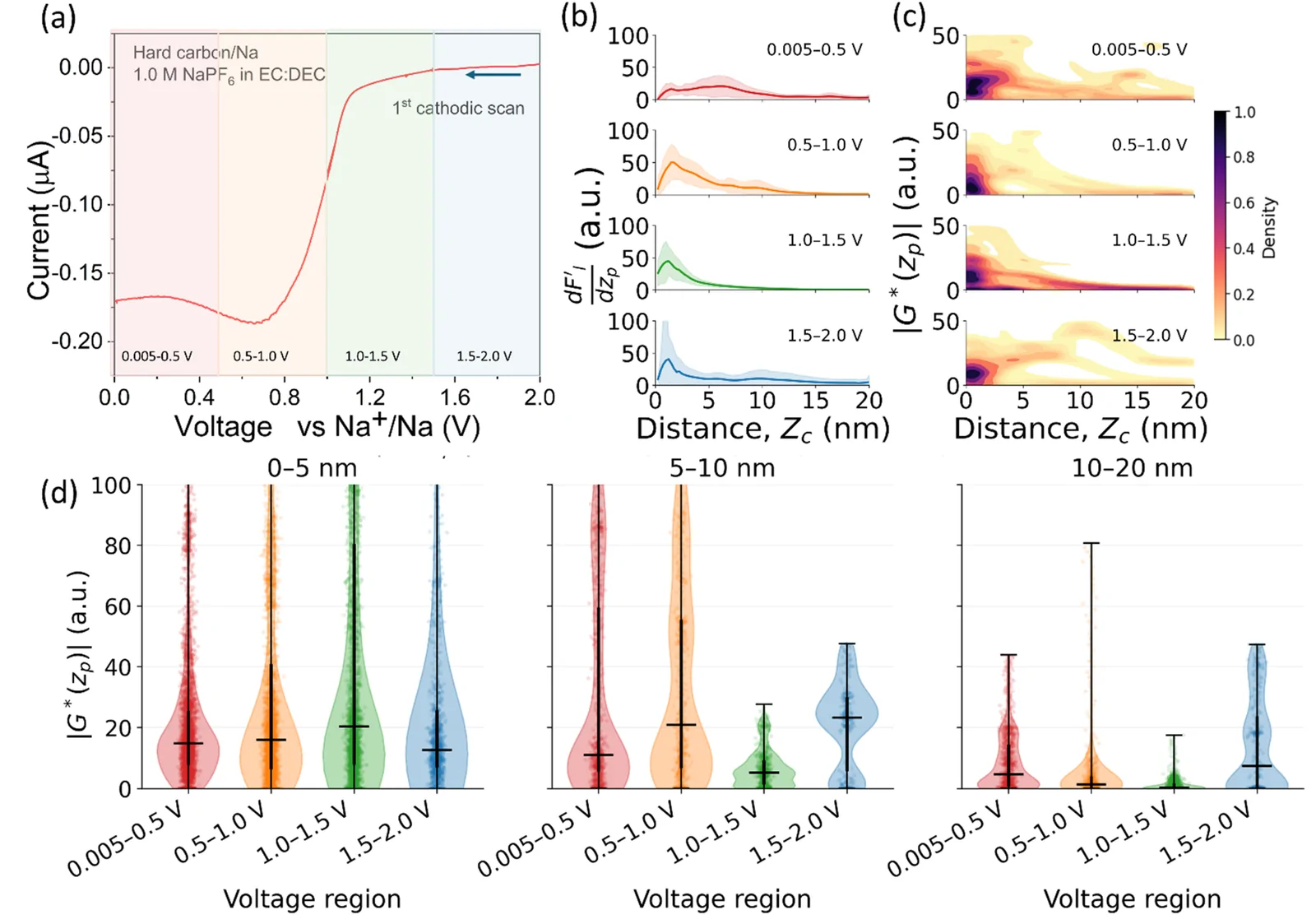
*Operando* 1D-NRM analysis of SEI evolution on a hard-carbon/Na system in 1.0 M NaPF_6_ in EC : DEC: (a) 1st cathodic or sodiation profile obtained from cyclic voltammetry at 1 mV s^−1^ highlighting the voltage regions used for further analysis, (b) depth-resolved 
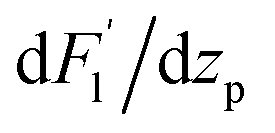
 profiles showing the evolution of the elastic response (mean ± CI), (c) Kernel density estimation (KDE) maps of the magnitude of the complex shear response |G*(*z*_p_)| as a function of depth and voltage and (d) violin plots of |G*(*z*_p_)| distributions for 0–5 nm, 5–10 nm and 10–20 nm depth intervals.

To probe SEI formation on real hard-carbon electrodes (HC) with high surface roughness, *operando* EC-NRM measurements were performed on an HC *vs.* Na system during initial sodiation using cyclic voltammetry from open circuit voltage (OCV) to 0.005 V *vs.* Na/Na^+^ in 1.0 M NaPF_6_ in EC : DEC (1 : 1 v/v) at 1.0 mV s^−1^ (Fig. 4a). The depth-resolved lateral force derivative (
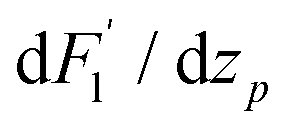
), extracted from the local referencing approach, is shown in Fig. 4b. The voltage range was divided into distinct regions corresponding to different stages of electrolyte reduction and SEI formation. The solid lines represent the mean profiles obtained by averaging all individual curves measured at each location in the voltage regions, while the shaded regions represent the 95% confidence interval (CI) of the mean, reflecting the statistical uncertainty of the ensemble-average response. The averaged profiles illustrate the variation of the local shear response as a function of the tip–surface distance *z*_p_. From Fig. 4b, it is clear that at large *z*_p_, *i.e.*, when the tip is away from the electrode surface, no lateral shear response is detected. As the tip approaches the surface, it begins to interact with the outer regions of the SEI and the measured response increases progressively with decreasing tip-surface distance. This increase reflects the gradual engagement of the probe with the interphase as it penetrates across the SEI from the outer soft organic to the inner stiff regions and provides an integrated measure of the SEI thickness across different electrochemical states.

For hard-carbon electrodes, the onset and evolution of the response vary with the voltage regions (Fig. 4b). However, a pronounced response is observed within the first few nanometres from the surface, followed by a gradual decay with increasing distance from the electrode surface across all voltage regions. At higher potentials (1.5–2.0 V), the response is weak at the far regions and confined to the near-surface region, indicating the absence of a well-developed interphase. As the potential decreases to 1.0–1.5 V, the onset of the interaction begins below 15 nm; the response gradually increases until the distance reaches ∼2 nm and then drops down, indicating the restructuring of the SEI. In the 0.5–1.0 V region, the onset of the interaction shifts to larger distances, suggesting the formation of a progressively thicker SEI. The gradual increase in lateral force response reflects the formation of soft skin-like or mechanically compliant outer regions and a transition towards denser or stiffer domains closer to the electrode surface, *i.e.*, below 5 nm. However, in the very low potential region 0.005–0.5 V, the onset of interaction begins at even at larger distances (20–30 nm) and the stiffness near the electrode surface decreases, which indicates the formation of a thicker and stable heterogeneous SEI. The response on hard carbon is distributed over a broad range compared to the well-defined peaks that indicate sharp transitions between layers in model flat systems. The observation of a high NRM response near the electrode surface (0–5 nm) indicates the presence of mechanically stiffer SEI domains, while the mixed response at the intermediate depths (5-10 nm) reflects the coexistence of softer and stiffer components within the interphase.

The two-dimensional kernel density (2D-KDE) maps^29^ of the absolute value of the complex shear modulus |G*(*z*_p_)| as a function of tip-penetration distance, *z*_p_, further illustrate the spatial distribution of the mechanically distinct SEI domains (Fig. 4c). The KDE maps represent the continuous probability density in |G*(*z*_p_)| in *z*_p_ space, where darker regions correspond to higher population density. These maps reveal distinct mechanical populations across voltages. In the higher voltage region, the KDE is concentrated near low response values and shallow depths, indicating mixed and limited interphase development. As the potential decreases, the distribution broadens both in response and depth, reflecting the emergence of heterogeneous SEI components. In low voltage regions, the KDE exhibits a pronounced spread, with contributions at higher response and SEI thickness, consistent with the formation of a thicker and structurally complex interphase.

The depth-dependent viscoelastic response and its redistribution is further quantified using “violin plots” across the depth intervals (0–5 nm, 5–10 nm and 10–20 nm) (Fig. 4c). In these plots, the width of the violin at a given value reflects the probability density of the data, *i.e.*, wider regions correspond to a higher concentration of the measurements. In the higher voltage region (1.5–2.0 V), at the outer SEI (5–10 and 10–20 nm), the distributions exhibit clear multimodal features, reflecting the heterogeneous nature of the hard-carbon surface and the presence of varying local environments. However, in the near-surface region (0–5 nm), the violin remains relatively narrow and predominantly uniform, indicating limited interphase development and a response largely related to the underlying electrode. At intermediate potentials (0.5–1.0 V), the response becomes more pronounced in all depth regions. In particular, at 0–5 nm, the distribution shifts towards higher response values, indicating the formation of an inner SEI. At 5–10 nm, the violin plots broaden significantly and begin to exhibit multimodality. This suggests the coexistence of multiple mechanical populations associated with a transitioning interphase. At 10–20 nm, the violin plot reveals the development of a more extended outer SEI layer. The low voltage region (0.005–0.5 V) shows a higher response value at 0–5 nm, which is consistent with a mechanically robust inner SEI. At intermediate depths, distinct multiple peaks are observed, indicating the presence of overlapping mechanical domains. In the outer SEI region (10–20 nm), the widespread and increased population reflects the formation of a thick heterogenous outer SEI composed of softer and more compliant components.

Overall, Fig. 4 demonstrates that the 1D-NRM approach enables reliable, depth-resolved and statistically robust characterization of SEI nanomechanics on realistic hard-carbon electrodes. The combined use of mean profiles, KDE maps and violin plots reveals not only the evolution of a viscoelastic response with voltage, but also the emergence and redistribution of heterogeneous nanomechanical populations across depths. These results confirm that the method effectively decouples nanoscale interphase properties from microscale surface roughness and provides a comprehensive framework to probe complex electrochemical interphases *operando*.

The observed evolution of the depth-dependent nanomechanical response in real battery electrodes with a rough surface through NRM is broadly consistent with the reports on SEI formation in sodium-ion systems, which describe an initial formation of an organic-rich outer SEI and an inorganic-rich inner layer based on compositional depth profiling methods such as hard X-ray photoelectron spectroscopy (HAXPES) and time-of-flight secondary ion mass spectrometry (ToF-SIMS^30^). This aligns with the transition from initial electrolyte-assisted decomposition to the formation of an extended, organic-rich outer SEI and a denser inorganic-rich inner layer. However, NRM measurements directly resolve the spatial distribution and dynamic redistribution of these mechanically distinct domains across depths, revealing the inherent heterogeneity of the SEI. This heterogeneity remains typically obscured in conventional measurements where the response reflects the mass–thickness variations integrated along the measurement direction, *i.e.*, effectively averaging contributions from multiple depths.

## 6 Conclusions

Here we report a novel approach based on AFM and the nanorheology microscopy (NRM) principle, allowing probing of the nanoscale mechanical properties of nanoscale SEI layers of real-life battery electrodes that have micrometre-scale roughness. We demonstrate that the NRM response is largely independent of the AFM tip radius within a reasonable range, therefore eliminating one of the most difficult parameters in nanomechanical studies *via* AFM and allowing true quantification of the nanorheological properties of the SEI with nm-scale depth resolution. By referencing the force–distance maps to the solid–solid–solid contact we can observe the dynamics of SEI growth on hard-carbon negative electrodes in half-cell Na-ion batteries *operando* while providing a depth resolution of a few nanometres. Furthermore, by probing the areas with an approximately 50 nm lateral step size, we achieve high quality informative statistical analysis of both the vertical and lateral variability of these electrodes.

## Conflicts of interest

All authors have no interests to declare.

## Data Availability

The authors have all experimental data available on the readers' request *via*o.kolosov@lancaster.ac.uk.
